# Hsp60 and IL-8 axis promotes apoptosis resistance in cancer

**DOI:** 10.1038/s41416-019-0617-0

**Published:** 2019-11-01

**Authors:** Sandeep Kumar, Jordan O’Malley, Ajay Kumar Chaudhary, Joseph R. Inigo, Neelu Yadav, Rahul Kumar, Dhyan Chandra

**Affiliations:** 1 Department of Pharmacology and Therapeutics, Roswell Park Comprehensive Cancer Center, Buffalo, NY USA; 2 Present Address: Department of Surgery, Division of Surgical Oncology, University of Illinois at Chicago, Chicago, IL 60612 USA

**Keywords:** Cancer therapeutic resistance, Cancer therapeutic resistance

## Abstract

**Background:**

Interleukin-8 (IL-8) and heat shock protein 60 (Hsp60) play crucial roles in cell survival and maintenance of cellular homoeostasis. However, cross talks between these two proteins are not defined.

**Methods:**

IL-8 expression in tumour tissue sections was analysed by immunohistochemistry. IL-8 expression and release in cancer cells was quantified using enzyme-linked immunosorbent assay (ELISA). Apoptosis was quantified using caspase activity and Annexin-V/PI staining.

**Results:**

We observed IL-8 release from cancer cells in response to histone deacetylase inhibitor, apicidin (Api), and non-competitive inhibitor of the sarco/endoplasmic reticulum Ca^2+^ ATPase, thapsigargin (TG). IL-8 release was increased upon TG-treatment. TG-induced IL-8 expression was reduced in the presence of Api in Bax-dependent manner. Increased apoptosis was associated with decreased IL-8 expression in response to combined treatment of TG and Api. TG and Api combination induced caspase-8 and caspase-9 dependent apoptosis. *Hsp60* knockdown abrogated IL-8 expression induced by Api, TG, and their combination. The level of TGF-β, an upstream regulator of IL-8, was decreased upon *Hsp60*-silencing. Knocking down *Hsp60* decreased IL-8 expression and its release in prostate cancer cell xenograft tumours in SCID mice.

**Conclusion:**

This study describes the underlying mechanism associated with apoptosis resistance mediated via Hsp60-IL-8 axis in cancer.

## Background

Drug resistance in malignant diseases is frequently observed and is an important concern for cancer treatment in the clinics. One of the main contributing factors for drug resistance is the evasion of apoptosis by tumour cells. Resistance to apoptosis is an accumulative outcome of several factors present within tumour microenvironment (TME). Cytokines and chemokines are important components of TME.^1^ Interleukin-8 (IL-8) or CXCL8 (C-X-C motif ligand 8) is a member of the chemokine family and associates with angiogenesis and endothelial cell migration.^2,3^ IL-8 participates in various cellular processes in cancer causing increased tumour progression and angiogenesis. Considering the important role of IL-8, it has been suggested as a biomarker to predict recurrence and overall survival of patients with non-small-cell lung cancer.^4^ Indeed, overexpression of IL-8 has been associated with many human tumours, including colorectal cancer (CRC) leading to poor prognosis.^5,6^ Since IL-8 promotes tumour growth, metastasis and angiogenesis, IL-8 could be an important therapeutic target in CRC.^7^ Overexpression of IL-8 renders colon cancer cells resistant to oxaliplatin while IL-8 silencing sensitises colon cancer cells to oxaliplatin.^7^ IL-8 is produced by activated endothelial cells in varieties of cancer types.^8^ In gastric carcinoma cells, IL-8 expression is associated with growth and vascularity of tumours.^9,10^ In colon cancer, constitutive expression of IL-8 correlates with metastatic potential and development of distant metastases.^11,12^ Pronounced release of IL-8 and CXC-family chemokines is reported from various types of cancer cell lines including prostate cancer cells.^13^ Transforming growth factor-β (TGF-β), a multifunctional cytokine acts upstream of IL-8 and regulates its function in prostate cancer.^14^ However, mechanisms of IL-8 mediated cellular signalling and its implication in cancer cell survival needs further characterisation.

In addition to IL-8, heat shock proteins (Hsps) or stress proteins have also been associated with TME and poor prognosis in various types of cancer.^15^ Heat shock protein 60 (Hsp60) expression levels correlates with cervix and colon carcinogenesis.^16,17^ Hsp60 is a key chaperonin, plays an essential role in the transport and folding of mitochondrial proteins, and is increased in different types of cancer. Hsp60 is overexpressed in prostate tumours and is strongly associated with prognostic clinical parameters.^18^ Hsp60 expression in prostate cancer was correlated with biochemical recurrence.^19^ Similarly, Hsp60 expression associates with tumour differentiation and stages in colorectal cancer.^20^ Exposure to extracellular Hsp60 renders the human lymphoma cell line U937 resistant to oxidative damage-induced apoptosis.^21^ Hsp60 also restrains p53 function by stabilising the antiapoptotic protein survivin, thus promoting cell proliferation.^22^ These findings provide strong relevance that the overexpression of IL-8 and Hsp60 could play critical role in maintaining TME, growth and invasion of cancer cells. Thus, we propose that inhibition of IL-8 and Hsp60 axis may attenuate cancer cell growth and survival.

For the first time, we report that Hsp60 regulates IL-8 production and release both in vitro cell culture and in vivo tumour xenografts models. We observed that thapsigargin (TG), a non-competitive inhibitor of the sarco/endoplasmic reticulum Ca^2+^ ATPase, induced robust expression of IL-8, which can be associated with IL-8 mediated cancer cell survival. A nanomolar combination of TG with histone deacetylase inhibitor apicidin (Api) downregulated IL-8 expression and induced apoptosis in colon and prostate cancer cells. Knockdown of *caspase-8* and *caspase-9* significantly decreased effector caspase-3 activity induced by combined treatment of TG and Api. Interestingly, reconstitution of Bax in cancer cells decreased IL-8 levels and knockout of *Bax* in cancer cells upregulated IL-8 expression and its release. IL-8 release was drastically reduced upon *Hsp60* knockdown. The PC-3 prostate cancer cell xenograft study utilising *Hsp60*-knockdown and mock PC-3 cells in SCID mice demonstrated down-regulation of IL-8 in serum in *Hsp60* knockdown xenografts. Collectively, our results provide a novel insight on IL-8 regulation by Hsp60 in cancer cells.

## Materials and methods

### Cell lines and reagents

Cell lines HCT116 wild type (WT) and HCT116 Bax^−/−^, androgen-dependent LNCaP cells and androgen-independent cell lines (DU145 Mock, DU145 Bax reconstituted and PC-3 cells) were either provided by Drs B. Vogelstein and Peter Daniel or purchased from American type culture collection.^23,24^ All cells were cultured using their respective medium and maintained at 37 °C in a humidified atmosphere in the presence of 5% CO_2_. Antibodies and sources are: IL-8 (Santa Cruz Biotechnology, Inc. Dallas, TX, USA, Cat # sc-8427); Caspase-8 (Enzo Life Sciences, Inc., Farmingdale, NY, USA, Cat # ALX-804-242-C100); Caspase-9 (Cell Signaling Technology, Danvers, MA, USA, Cat # 9502), beta actin (Santa Cruz Biotechnology, Inc., Cat # sc-47778 HRP) and Hsp60 (Millipore-Sigma, Burlington, MA, USA, Cat # MAB3514). All reagents used in this study were of highest grade of purity.

### Treatment

Apicidin (Api) and Thapsigargin (TG) were purchased from Enzo Life Sciences, Inc. All cell lines were treated with either 200 nM Api or 500 nM TG alone or in combination. Cells (2.0 × 10^5^ cells/ml) were seeded on to six-well cell culture plate for 24 h and treated with different drugs alone or in combination.

### TCGA analysis

The TCGA colon adenocarcinoma (COAD) and colorectal adenocarcinoma (COREAD) datasets (version 2016-08-16) were retrieved from the UCSC Xena Browser. The polyA+ IlluminaHiSeq gene expression dataset was downloaded in its normalised format. RNA-seq values were grouped between primary tumour (PT) and matched non-tumour (MN) samples. The average transcript reads were calculated for each group. The RPKM method was used to quantify gene expression from RNA sequencing data by normalising for total read length and the number of sequencing reads. A student’s *t*-test was performed between matched normal and primary tumour groups.

### Isolation of whole cell lysate

These processes were carried out according to the protocol described earlier.^25^

### Western blotting

Whole-cell lysate (WCL) was prepared using NP-40-HEPES lysis buffer. WCL was separated by precast 26 well, 4–20% sodium dodecyl sulfate-polyacrylamide gel electrophoresis (SDS-PAGE) procured from Bio-Rad (Hercules, CA, USA) and transferred on to nitrocellulose membrane (Bio-Rad, Hercules, CA). Membranes were blocked in 5% non-fat milk for 30 min and washed with PBS-T (1X PBS and 0.1% Tween 20) and further incubated with respective primary antibody. Anti-mouse or anti-rabbit horseradish peroxidase-conjugated antibodies (Amersham Pharmacia Biotech, Piscataway, NJ, USA) were used as secondary antibody. After washing with PBS-T, proteins were detected using Clarity chemiluminescent reagent (Bio-Rad) and X-Ray films (ASI, Fort Lauderdale, FL, USA). Membranes were stripped using stripping buffer and probed with HRP conjugated beta-actin antibody.

### *Caspase-8, caspase-9, Hsp60* and *IL-8* knockdown using lentiviral shRNAs

Lentiviral shRNAs were procured from the Gene Modulation Services Resource (GMSR) core of Roswell Park Comprehensive Cancer Center, for knocking down expression of various genes in cancer cells. The shRNA sequences were: *caspase-8* (5′-GACTTCAGCAGAAATCTTT-3′), *caspase-9* (5′-CCAGGCAGCTGATCATAGA-3′), *Hsp60 or HSPD1* (5′-GCTATATTTCTCCATACTTTA-3′), and *CXCL8 or IL-8* (5′-TGCTATTTGTATATTCTCC-3′). Briefly, cells were seeded (5 × 10^4^ cells) per well of 6-well plates. After 24 h, polybrene (8 µg/ml) was added to the media. After 1 h, mock shRNA or gene specific shRNA (*caspase-8, caspase-9, IL-8* and *Hsp60*) lentiviral particles were added at MOI of 3. After 48 h of transduction, media was replaced with fresh media containing 1 µg/ml puromycin for selection of transduced cells. Knockdown of targeted gene was confirmed using immunoblotting or ELISA.

### Bright field microscopy

Cellular morphological changes induced by treatment as well as in untreated control were assessed by bright field microscopy. Images were captured without refreshing medium by an inverted microscope (Olympus, Waltham, MA, USA).

### ELISA assay

Estimation of IL-8 and TGF-β1 levels was carried out using commercially available kits (eBiosciences, San Diego, CA, USA, and Ray Biotech, Peachtree Corners, GA, USA respectively). For data normalisation, the value of cytokines in lysates or supernatant was divided by protein concentration of lysates.

### Caspase-3 activities

Caspase-3 activity was quantified using a fluorometric method described earlier.^26^ In a 96-well plate, cell lysates prepared in NP-40-HEPES lysis buffer were incubated with DEVD-AFC (caspase-3 substrate) at 37 °C for 90 min in caspase activity assay buffer (50 mM HEPES pH 7.4, 150 mM NaCl, 0.5% CHAPS, 1 mM EDTA, 1mM DTT, 25% glycerol). Fluorescence intensity was detected using a Synergy microplate reader at excitation and emission wavelengths of 400 nm and 508 nm, respectively. Arbitrary fluorescence units were normalised with protein content of cell lysates and represented as fold change compared to control groups. Protein concentration of WCL was determined by microBCA kit (Thermo Fisher Scientific, Grand Island, NY, USA) where bovine serum albumin (BSA) was taken as a standard. The fluorescence obtained for caspase-3 was normalised by their respective protein concentration. The results were presented in the fold change when compared to control.

### Annexin/PI staining

Cells were treated with various anticancer agents or with vehicle for various time periods followed by staining with annexin-V-Alexa flour 488/PI kit (Invitrogen, Waltham, MA, USA) according to the manufacturer’s instructions. The stained cells were analysed by flow cytometry (LSRIIA, BD Biosciences, San Jose, CA, USA) collecting 10 000 events. Data were analysed using Win List 3D 7.1 software.

### Mice and PC-3 xenograft study

Male SCID (C.B-Igh-1^b^/IcrTac-Prkdc^scid^) mice (6-weeks-old; ~25 g body weight) were purchased from an in-house breeding colony maintained by Roswell Park Comprehensive Cancer Center Laboratory Animal Shared Resource. Mice were housed in standard ventilated cages, up to five animals per cage, with commercially available laboratory animal cages bedding at 24 ± 1 °C in a light-controlled room (light: 6:00–18:00 h, dark: 18:00–6:00 h) and a relative humidity of 55% ± 5%. Mice were given an ad libitum supply of filtered pathogen-free air, food, and water. Mice were acclimatised for a week and prior to implantation of tumours during evening hours. Animals were anaesthetised by inhalation of isoflurane mixed with oxygen using an isoflurane vaporiser. *Mock* or *Hsp60* knockdown PC-3 cells (1 × 10^6^ cells) were re-suspended in ice cold PBS and mixed with matrigel (50:50 v/v) and injected subcutaneously in both flanks of SCID mice (five mice/group) under anaesthesia using 27G needle inside a biosafety cabinet. Isoflurane inhalation anaesthesia was chosen because of its faster induction and recovery, minimal effect on cardiovascular function and negligible metabolism. Animals were monitored daily after implantation of tumour. Tumours size in animals were measured twice weekly using callipers. Body weight of animals were measured twice a week. Once mock PC-3 xenografts reached 2 cm in cumulative diameter, mice were killed by carbon dioxide inhalation followed by cardiac puncture for exsanguination of blood for serum and serum kept at −80 °C until further use. Tumours were harvested, cut into small pieces and fixed in 10% formalin (at least for 48 h) followed by 70% ethanol for a week. After fixation, tumours were placed into cassettes for processing and embedding in paraffin blocks. For immunohistochemistry, paraffin-embedded tumour tissues were sectioned (3–5 μm) and stained with haematoxylin and eosin (H&E). To determine IL-8 expression in xenograft tissues, we performed immunohistochemistry and viewed under a microscope (Olympus, Waltham, MA, USA). Serum IL-8 levels were determined by human IL-8 specific ELISA.

### Immunohistochemistry

For tissue section staining, following de-paraffinisation and dehydration, slides were incubated in 3% hydrogen peroxide to block endogenous peroxidase activity. For antigen retrieval, slides were incubated in 10 mM citrate buffer, pH 6.0, for 15 min in a microwave oven. Then slides were sequentially incubated in blocking solution (10% goat serum in PBS, 30 min), primary antibody (anti-IL-8; 1:1,000 for overnight at 4 °C), and secondary antibody (goat anti-rabbit IgG conjugated to HRP; 1:4,000 for 1 h at RT). Slides were developed with DAB peroxidase substrate kit (Vector Labs, Burlingame, CA, USA).

### Statistical analysis

Significant differences between means of presented data including studies related to PC-3 cell xenografts were assessed using two groups Student’s *t*-test and multiple groups Bonferroni multiple comparison test by Graph Pad Prism 5.0. Significance was denoted as compared to control, unless otherwise indicated.

## Results

### IL-8 is overexpressed in colorectal cancers

**Fig. 1:**
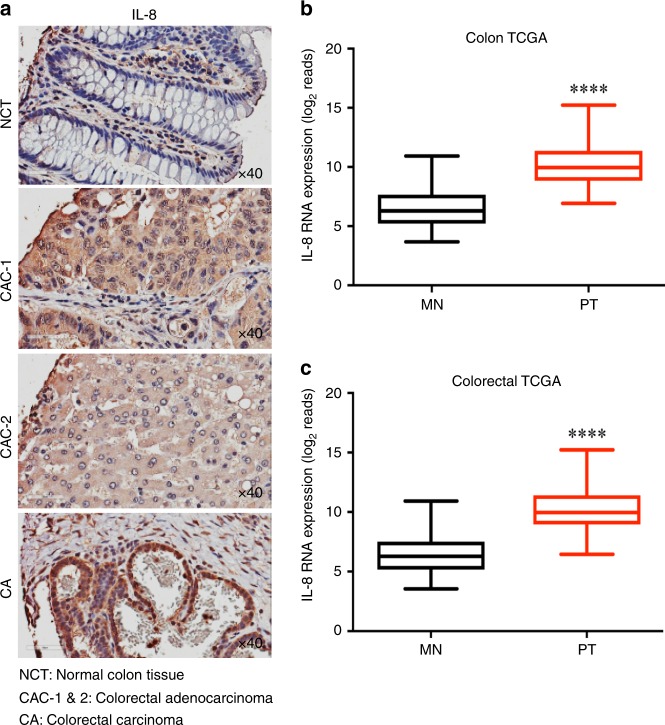
IL-8 is overexpressed in colon cancers. **a** Immunohistochemistry (IHC) analysis of IL-8 expression in normal colon, human colorectal adenocarcinoma, and colorectal carcinoma tissue samples using tissue microarray (TMA) sections. Images were scanned using Aperio ImageScope. **b**, **c** TCGA analysis for IL-8 transcript levels in colon (*n* = 26) and colorectal cancer (*n* = 34). MN matched non-tumour tissues, PT primary tumour tissues. Data is presented as mean ± SD. ****p < 0.05 vs respective controls

The chemokine, IL-8 plays an important role in tumour growth, angiogenesis, and metastasis.^27,28^ To understand the expression level of IL-8 in human cancer, we used tumour microarray (TMA) of multiple cancers and performed immunohistochemistry (IHC) staining. The multi-cancer TMA showed increased expression of IL-8 in various cancers including colorectal adenocarcinoma (CAC-1 and CAC-2), and colorectal carcinoma compared to normal colon tissue (Fig. 1a). We further estimated *IL-8* mRNA expression in TCGA-colon and TCGA-colorectal cancer database and observed overexpression of *IL-8* levels in both cancer types compared to matched non-tumour specimens (Fig. 1b, c). Together, IHC and TCGA database analysis suggests that IL-8 is overexpressed in colorectal cancer.

### Combination of Api and TG enhances apoptosis and reduces IL-8 expression in cancer cells

**Fig. 2:**
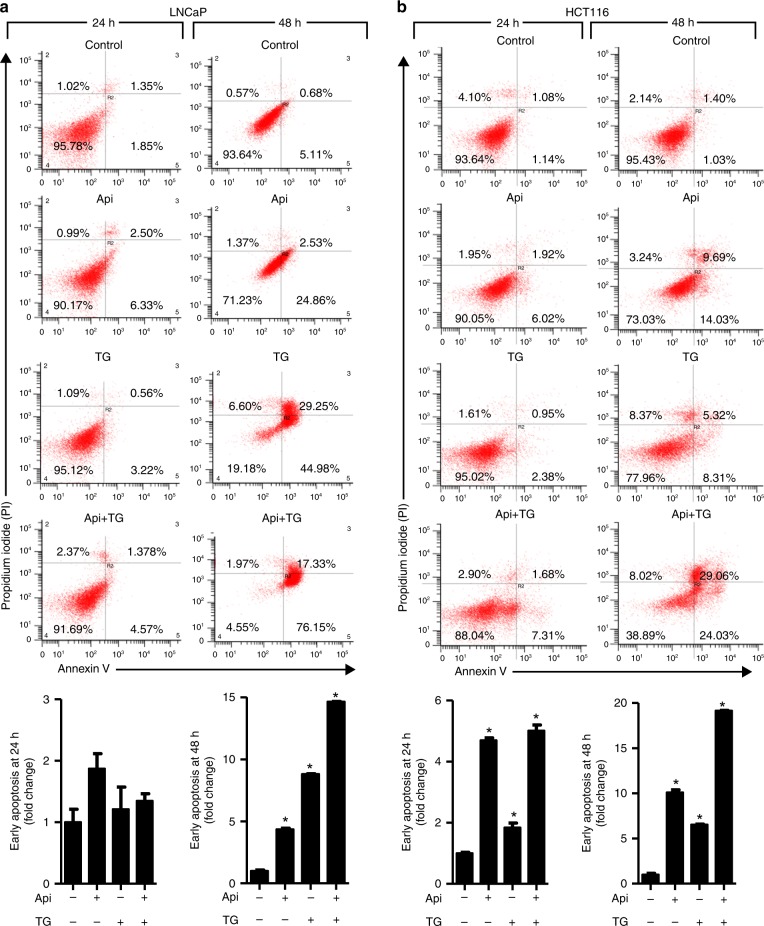
Combination of Api and TG induces apoptosis. **a**, **b** Flow cytometry analysis of apoptosis in LNCaP and HCT-116 cells using Annexin-V and PI staining. A total 10,000 events were captured, and result was analysed by Win List software. Data are mean ± SD (*n* = 3). *p < 0.05 vs respective controls

**Fig. 3:**
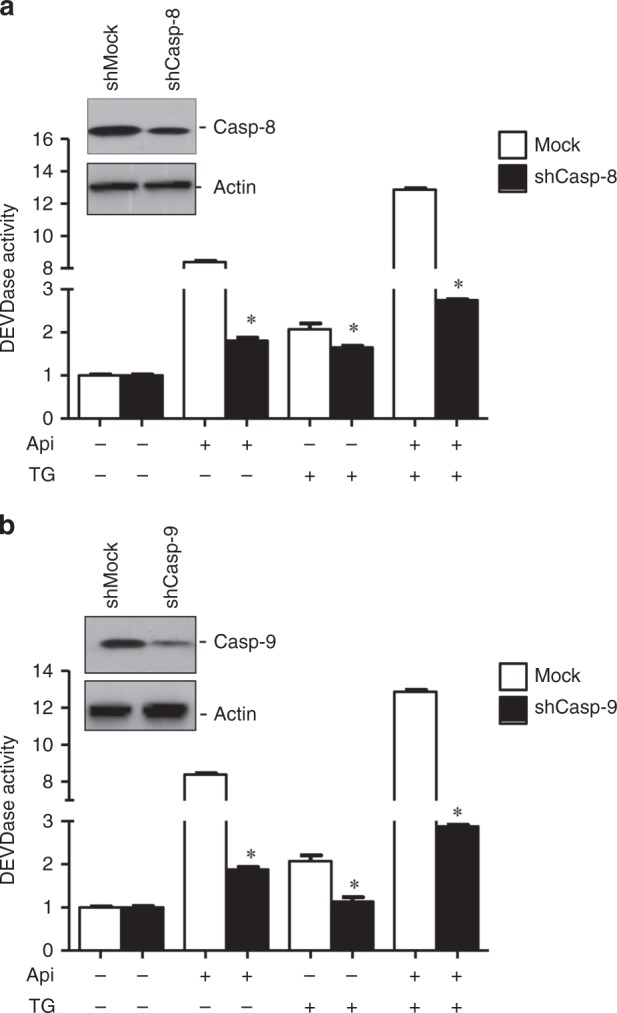
Combination of Api and TG induces caspase activity. Caspase-8 (Casp-8) and caspase-9 (Casp-9) were silenced in HCT116 cells using shRNA. Mock shRNA was used as control. **a**, **b** Caspase-3 activities were measured in mock and *caspase-8* and *caspase-9* silenced cells following treatment with Api or TG alone or their combination. Whole cell lysates were used to analyse caspase-8 and caspase-9 knockdown using western blotting. Actin serves as a loading control. Data are mean ± SD (*n* = 3). *p < 0.05 vs respective controls/groups

**Fig. 4:**
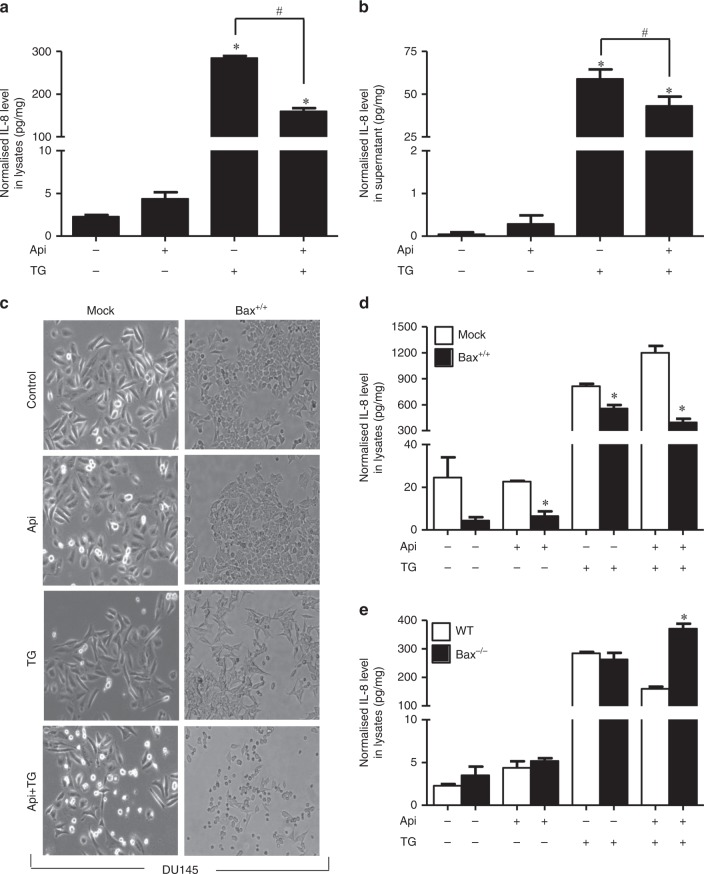
Bax and IL-8 are inversely correlated in prostate and colon cancer cells. HCT116 cells were treated with Api and TG alone and in combination. The level of IL-8 was determined in lysate (**a**) and supernatant (**b**) using ELISA. Mock and *Bax* reconstituted DU145 cells were treated with Api and TG alone and in combination. (**c**) Bright field microscopy images of control, Api, TG and Api plus TG treated mock and *Bax* reconstituted DU145 cells. **d** IL-8 was quantified by ELISA using DU145 whole cell lysates. **e** IL-8 was quantified using whole cell lysates isolated from HCT116 WT and *Bax* knockout cells. Data are mean ± SD (*n* = 3). *p < 0.05 vs respective controls

Since we observed that IL-8 is overexpressed in cancer, we next determined whether/how IL-8 is involved in apoptosis resistance in colon and prostate cancer cells. Thus, we selected two pharmacological inhibitors, Api and TG, which are known to induce autocrine release of IL-8 from cells.^29,30^ Drug-induced IL-8 signalling promotes resistance to apoptosis in LNCaP and PC-3 prostate cancer cells.^31^ To determine the importance of IL-8 release on apoptosis, we treated cancer cells with Api and TG alone or in a combination. Annexin/PI staining suggests increased cell death in a time-dependent manner induced by Api and TG in prostate and colon cancer cells (Fig. 2a, b). We have used very low doses of Api, or TG as compared to earlier studies^32–34^ in concluding that this combination does not show overt toxicity. Anticancer activities in response to the exposure of these drugs were more effective at later time points (48 h) in both cancer cell types. Apoptosis is mediated by several factors including activation of caspases.^25,35^ Caspases are involved in apoptosis and have been sub classified by their mechanism of action as initiator caspases such as caspase-8 and -9 or executioner caspases such as caspase-3, -6, and -7.^36^ To determine whether IL-8 mediated apoptosis requires caspases, we treated mock, *caspase-8* and *caspase-9* knockdown HCT116 colon cancer cells with TG and Api alone or their combination. We observed that combination treatment of Api and TG significantly induced caspase-3 activity in *mock* HCT116 cells (Fig. 3a, b). *Caspase-8* and *caspase-9* knockdown in HCT116 cells inhibited caspase-3 activity confirming the involvement of both of the initiator caspases in Api and TG combination treatment-induced apoptotic cell death in cancer cells (Fig. 3a, b). Since Api and TG are known to induce IL-8, we determined expression of IL-8 upon treatment of TG and Api alone or in a combination in cancer cells. We observed that treatment of TG induces robust IL-8 production and release whereas Api-induced IL-8 level and release was not as prominent as TG (Fig. 4a, b). Interestingly, combination of Api with TG abrogates IL-8 production and release induced by TG. Taken together, our results suggest that increased autocrine release of IL-8 is associated with decreased caspase activation and apoptosis.

### Bax downregulates IL-8 expression and release

Pro-apoptotic protein Bax plays a critical role in the permeabilisation of outer mitochondrial membrane leading to cytochrome c release, caspase activation and apoptosis.^37^ To address the importance of Bax in IL-8 mediated apoptosis, we used isogenic colon cancer cell lines HCT116 wild type (WT) and Bax^−/−^ as well as isogenic prostate cancer cell line DU145 mock and Bax reconstituted (DU145 Bax^+/+^) cells. We observed that Bax reconstitution induced a higher level of cell death in response to Api and TG combination (Fig. 4c). It has been reported that endothelial cells incubated with IL-8 promotes higher levels of Bcl-2:Bax ratios,^2^ and therefore, we determined interrelation of Bax and IL-8 in prostate and colon cancer cells. We observed that IL-8 expression was significantly decreased upon reconstitution of Bax (Fig. 4d). We further studied the effect of Bax knockout on IL-8 expression in WT and Bax^−/−^ HCT116 cells. IL-8 expression was increased in the absence of Bax (Fig. 4e) in response to combined exposure of TG and Api. Collectively, Bax and IL-8 expression are inversely regulated in prostate and colon cancer cells.

### *Hsp60* knockdown inhibits TGF-β expression, an upstream regulator of IL-8

**Fig. 5:**
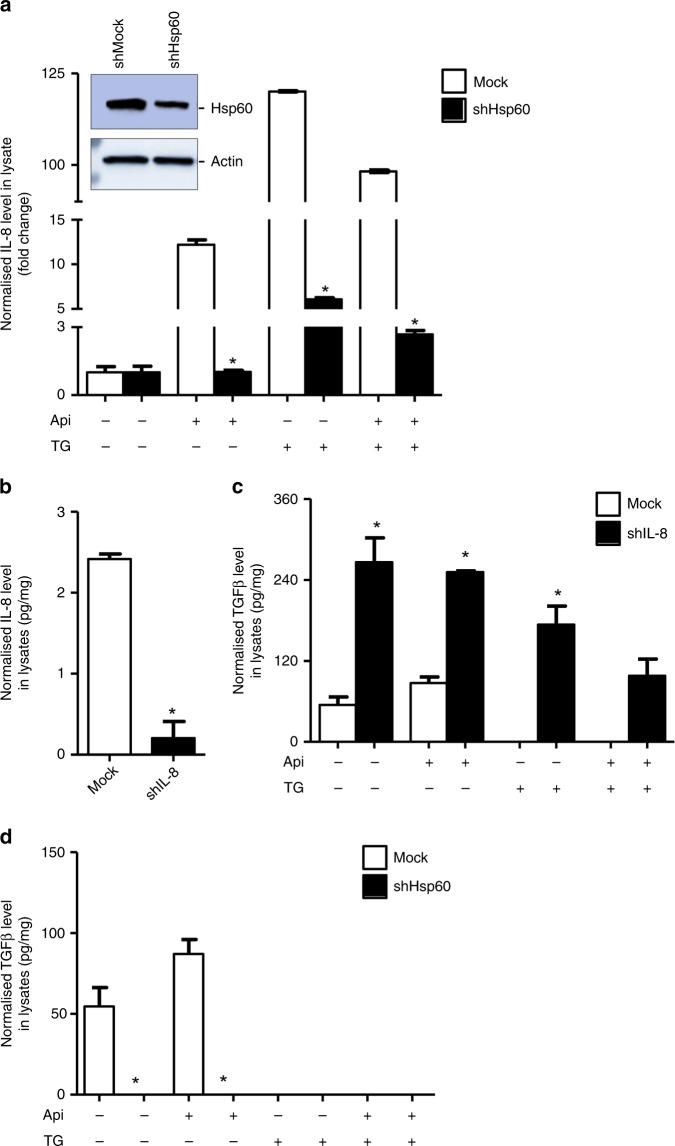
*Hsp60* knockdown inhibits TGF-β and IL-8 expression, whereas IL-8 enhances TGF-β. *Hsp60*-silenced and mock shRNA LNCaP cells were treated with Api, TG alone or in combination followed by IL-8 quantification using ELISA (**a**). Whole cell lysates were used to analyse Hsp60 knockdown using Western blotting. Actin serves as a loading control. **b** *IL-8* knockdown was confirmed by ELISA in cell lysate. **c** Estimation of TGF-β in cell lysates using ELISA in mock and *IL-8* knockdown LNCaP cells following treatment with Api or TG alone or their combination. **d** Estimation of TGF-β in cell lysates using ELISA in mock and *Hsp60* knockdown LNCaP cells following treatment with Api or TG alone or their combination. Data are mean ± SD (*n* = 3). *p < 0.05 vs respective controls/groups

Acute ablation of Hsp60 destabilises mitochondrial functions leading to increased expression of proapoptotic Bax and Bax-dependent apoptosis.^22^ Since IL-8 and Hsp60 inversely associate with Bax, we next determined effect of *Hsp60* knockdown on IL-8 expression. A robust decrease in IL-8 expression was observed upon *Hsp60*-silencing in LNCaP prostate cancer cells (Fig. 5a). TGF-β plays a central role in tumour management and regulates expressions of cytokines including IL-8.^14^ We determined the effect of *IL-8* knockdown on TGF-β expression and observed that *IL-8* knockdown promotes TGF-β expression (Fig. 5b, c). Since Hsp60 promotes IL-8 expression and TGF-β also regulates IL-8 levels, we next asked the question, whether Hsp60 is critical for TGF-β expression. We used mock and *Hsp60* knockdown LNCaP prostate cancer cells and treated with Api or TG alone or in combination. Decreased expression of TGF-β in TG alone or in combination of Api and TG was observed in control (mock) LNCaP cells, whereas TGF-β was not detectable in *Hsp60* knocked down LNCaP cells (Fig. 5d). Taken together, Hsp60 regulates IL-8 expression as well as its upstream regulator TGF-β in cancer cells.

### *Hsp60* knockdown inhibits IL-8 expression and release in vivo

**Fig. 6:**
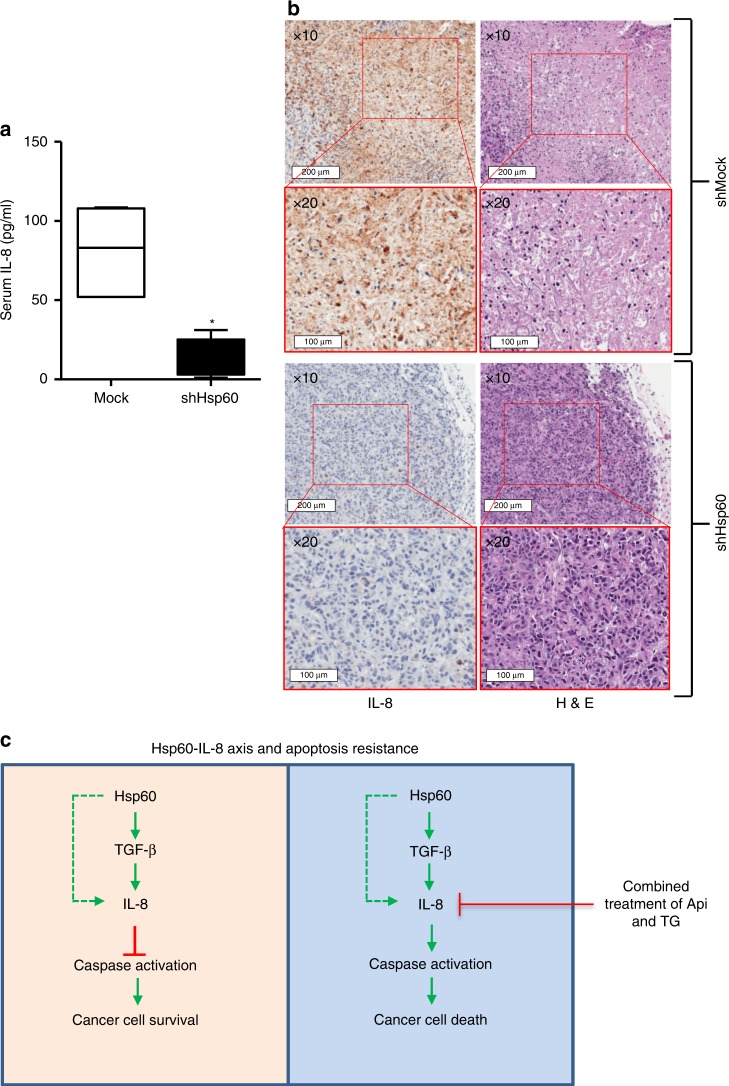
*Hsp60* knockdown inhibits IL-8 expression and release. **a** Estimation of IL-8 in serum of mock and *Hsp60* knockdown PC-3 cell xenografts using ELISA. **b** Immunohistochemistry of IL-8 in tumour sections of mock and *Hsp60* knock down xenografts. **c** Schematic diagram of Hsp60-IL-8 axis and its role in apoptosis resistance. Data are mean ± SD (*n* = 5). *p < 0.05 vs controls

The overexpression of Hsp60 associates with increased expression of anti-apoptotic proteins Bcl-xl and Bcl-2, and decreased expression of the pro-apoptotic protein, Bax.^38^ To understand the physiological relevance of our study, we next performed in vivo experiments to determine whether Hsp60 regulates IL-8 production and release in tumour cells in vivo. We subcutaneously grafted mock and *Hsp60* knockdown PC-3 prostate cancer cells in SCID mice. Human IL-8 levels in mouse serum of mock and *Hsp60* knockdown groups were subsequently estimated by human IL-8 specific ELISA. The levels of IL-8 in mouse serum were significantly decreased in *Hsp60* knockdown group compared to mock group (Fig. 6a). In the mock group (*n* = 5), levels of IL-8 in serum were 108.5, 83.0, 107.3, 52.0 and 52.0 pg/ml, respectively while in *Hsp60* knockdown group (*n* = 5), IL-8 levels were 1.01, 5.15, 19.25, 18.55 and 31.24 pg/ml, respectively (Fig. 6a). We also determined IL-8 expression in tumours by IHC staining using human anti-IL-8 antibody. Concurrent with serum IL-8 results, expression of IL-8 was drastically downregulated in *Hsp60* knock down tumours (Fig. 6b). Taken together, these findings clearly indicate that Hsp60 regulates the IL-8 expression and its release in tumour cells.

## Discussion

Deregulated cytokine expression and signalling play a crucial role in developing drug resistance in cancer.^39^ Aberrant cytokine signalling induces proliferation of tumour cells and facilitates formation of stromal blood vessel networks, which promotes tumour progression and growth.^39,40^ We report that Hsp60 regulates IL-8 expression and release in colon and prostate cancer. Increased levels of IL-8 and Hsp60 are associated with enhanced resistance to apoptosis in cancer cells. Hsp60 either directly or via TGF-β upregulates IL-8 expression, which inhibits apoptosis leading to cancer cell survival. The combined treatment of Api and TG downregulates IL-8 expression causing increased caspase activation and cancer cell death (Fig. 6c).

Most of the chemotherapeutic drugs fail to induce efficient apoptosis in cancer cells. Our study provides the underlying mechanism on how autocrine release of IL-8 contributes to tumour growth and apoptosis resistance in cancer cells. Since, Api and TG are reported to induce autocrine release of IL-8,^29,30^ we used these two drugs alone or in combination to study the effect of IL-8 release on apoptosis in prostate and colon cancer cells. Drug-induced IL-8 upregulation has also been reported in dacarbazine treated melanoma cells, etoposide and mitomycin C treated human epithelial carcinoma cells, and doxorubicin treated human small cell lung cancer cells.^40^ Chemotherapy-induced IL-8 signalling promotes resistance in cancer cells via upregulation of prosurvival protein such as cellular FLICE-inhibitory protein (c-FLIP).^31^ Abrogation of TG-induced IL-8 expression and increased apoptosis in presence of Api suggests that, the combination of these two drugs may overcome IL-8-mediated cell survival and resistance. Apoptosis is an outcome of a diverse cascade of cellular events mediated by several molecules including caspases and pro-apoptotic proteins.^41,42^ The combination of TG and Api promotes caspase-8, caspase-9, and Bax mediated apoptosis. Cytokine-mediated Bax deficiency and consequent delayed apoptosis has already been reported in neutrophils.^43^ Our findings provide evidence that Bax downregulates IL-8 expression in colon and prostate cancer cells.

The anti-apoptotic mechanism of mitochondrial chaperone Hsp60 involves sequestration of Bax-containing complexes that may lead to the inhibition of apoptotic cell death.^22^ We observed a new role of Hsp60 in regulating IL-8 and cell survival in colon and prostate cancer. Api and TG combination disrupts Hsp60-IL-8 axis, and therefore, may overcome apoptosis resistance. Similar to our study, knockdown of *IL-8* has also been reported to increase sensitivity to cisplatin in platinum-sensitive cells and reversed platinum resistance in resistant cell lines.^44^ We demonstrated regulation of IL-8 production and release by Hsp60 in vitro and in vivo. To validate IL-8 release by tumour cells, we used immune deficient SCID mice to study regulation of IL-8 by Hsp60. Since we used PC-3 human prostate cancer cells for xenograft studies and determined IL-8 production by using anti-human IL-8 antibody, our results confirmed production and release of IL-8 by cancer cells (and not from murine cells) and its regulation by Hsp60. Increased IL-8 in serum associates with poor response to cancer therapeutics.^45^ IL-8 recruits pro-tumorigenic factors to remodel TME.^46^ IL-8 expression in cancer-associated fibroblasts (CAFs) also modulates TME leading increased NF-κB activation and chemoresistance in human gastric cancer.^47^ Thus, downregulation of IL-8 release in serum and decreased expression in tumour sections upon *Hsp60* knockdown PC-3 xenograft suggest that Hsp60 promotes tumour progression via IL-8-mediated TME remodelling. TGF-β signalling regulates IL-8 expression in cancer cells including human prostate cancer cells.^14^ Although regulation of IL-8 is contributed by TGF- β, exposure of Hsp60 causes elevated TGF-β expression in diseased conditions.^48,49^ Api-induced TGF-β was diminished by TG, suggesting that the combined exposure of Api and TG may overcome apoptotic resistance mediated by the IL-8-TGF-β axis. Interestingly, Hsp60 regulates both IL-8 and TGF-β pro-survival interleukins and acts as an upstream regulator of IL-8-TGF-β signalling axis.

In summary, we report that Hsp60 regulates IL-8 and TGF-β production and their release in cancer cells. Hsp60-mediated upregulation of IL-8 and TGF-β correlates with downregulation of caspase activities and inhibition of apoptotic cell death. Combined exposure of Api and TG may abrogate Hsp60-IL-8-TGF-β axis to overcome apoptotic resistance in cancer cells. IL-8 is an autocrine growth factor remodels TME and associates with disease recurrence in many types of cancer including in prostate and colon cancer.^13,50,51^ Increased expression of Hsp60 contributes tumour progression, apoptosis inhibition, modulation of TME, therapy resistance, and recurrence of many types of cancer.^19,52–58^ This study provides strong evidence of Hsp60 regulation of IL-8 expression and release, which plays critical role TME remodelling and therapeutic resistance in cancer. Therefore, targeting Hsp60-IL-8 axis could have a potential therapeutic outcome in colorectal and prostate malignancies.

## Data Availability

Summarised primary research data are presented in the paper. No publicly available dataset has been generated as part of this work.
